# Genetic architecture of inducible and constitutive metabolic profile related to drought resistance in qingke (Tibetan hulless barley)

**DOI:** 10.3389/fpls.2022.1076000

**Published:** 2022-12-06

**Authors:** Kuohai Yu, Lingling Wei, Hongjun Yuan, Weiqin Zhang, Xingquan Zeng, Bin Wang, Yulin Wang

**Affiliations:** ^1^ State Key Laboratory of Hulless Barley and Yak Germplasm Resources and Genetic Improvement, Lhasa, China; ^2^ Institute of Agricultural Research, Tibet Academy of Agricultural and Animal Husbandry Sciences, Lhasa, China; ^3^ Wuhan Metware Biotechnology Co., Ltd, Wuhan, China

**Keywords:** Tibetan hulless barley, drought resistance, mGWAS, inducible and constitutive accumulation, raffinose, flavonoids

## Abstract

Qingke (Tibetan hulless barley, *Hordeum vulgare* L. var. *nudum*) is the primary food crop on the Tibet Plateau, the long-term drought and other harsh environments makes qingke an important resource for the study of abiotic resistance. Here, we evaluated the drought sensitivity of 246 qingke varieties. Genome-wide association studies (GWAS) found that root-specific expressed gene *CYP84* may be involved in the regulation of drought resistance. Based on widely targeted metabolic profiling, we identified 2,769 metabolites in qingke leaves, of which 302 were significantly changed in response to drought stress, including 4-aminobutyric acid (GABA), proline, sucrose and raffinose. Unexpectedly, these drought-induced metabolites changed more violently in drought-sensitive qingkes, while the constitutive metabolites that had little response to drought stress, such as *C*-glycosylflavonoids and some amino acids, accumulated excessively in drought-resistant qingkes. Combined with metabolite-based genome-wide association study (mGWAS), a total of 1,006 metabolites under optimal condition and 1,031 metabolites under mild drought stress had significant associated loci. As a marker metabolite induced by drought stress, raffinose was significantly associated with two conservatively adjacent α-galactosidase genes, qRT-PCR suggests that these two genes may jointly regulate the raffinose content in qingke. Besides, as constituent metabolites with stable differences between drought-sensitive and drought-resistant qingkes, a class of *C*-glycosylflavonoids are simultaneously regulated by a UDP-glucosyltransferase gene. Overall, we performed GWAS for sensitivity and widely targeted metabolites during drought stress in qingke for the first time, which provides new insights into the response mechanism of plant drought stress and drought resistance breeding.

## Introduction

Drought is one of the most common stresses in crops, which directly leads to the decrease of photosynthesis, the accumulation of reactive oxygen species (ROS) and the damage of cell membrane, ultimately leads to significant reduction of yield and quality (Kapoor et al., 2020). It is of great theoretical and practical significance to identify drought resistant materials in important crops for mining their elite alleles of drought tolerance. Tibetan hulless barley (*H. vulgare L.* var. *nudum*) is cultivated barley in Tibet Plateau, and also known as “qingke”. Qingke exhibits strong adaptability to extreme environments and has become a typical representative of the evolution of plant adaptability (Zeng et al., 2018). Some stress-related genes have been cloned and utilized from qingke. For example, natural variation of phenylpropanoid content involved in UV-B protection in qingke was regulated by flavone *C*-pentosyltransferase, tyramine hydroxycinnamoyltransferase and MYB transcription factor genes (Zeng et al., 2020). Drought tolerance analysis shows that RNAi transgenic lines of two late embryogenesis abundant (*LEA*) family genes (*HVA1* and *Dhn6*) in qingke have a significantly reduced survival rate under drought stress, and the *HVA1* gene regulates the water loss rate of plants under drought stress by participating in vegetative growth (Liang et al., 2012).

Plants have evolved a variety of efficient strategies in response to drought stress, including osmotic pressure and antioxidant regulation. Under drought stress conditions, rice leaves accumulate a large amount of proline and soluble sugar, which rise sharply in the early stage of drought stress, and then slowly decrease after reaching the peak (Xiong et al., 2012). Drought stress also induced the accumulation of raffinose and its precursor galactinol as osmoprotectants in plant cells (Taji et al., 2002; Nishizawa et al., 2008). Li et al. found that maize with the raffinose synthase (RAFS) gene mutation lacked raffinose, has hyper-accumulation of galactinol, and was more sensitive to drought stress than wild type. However, overexpression of *ZmRAFS* gene in *Arabidopsis thaliana* promotes the hydrolysis of galactinol in leaves to produce *myo*-inositol to enhanced drought stress tolerance (Li et al., 2020). In addition, there are two kinds of antioxidant systems in plants to eliminate ROS accumulated by stress: enzymatic and non-enzymatic. The enzymatic components mainly include superoxide dismutase (SOD), catalase (CAT), peroxidase (POD), ascorbate peroxidase (APX). The non-enzymatic antioxidant system is composed of some reduced metabolites like ascorbic acid, glutathione (GSH), cytochrome, α-tocopherol and flavonoids, among which flavonoids show clear plant species specificity, suggesting its important role in the process of plant stress (Das and Roychoudhury, 2014; Hakan Cetinkaya, 2017). It is worth noting that the stability and bioactivity of flavonoids are regulated by glycosylation modification (Le Roy et al., 2016), while the contribution of flavonoids to drought resistance among varieties and its regulatory genes under drought stress remain largely unknown.

Although drought resistance relies heavily on the maintenance of cell osmotic pressure and ROS clearance involved by metabolites, the response patterns of these drought-resistance metabolites and their regulatory genes are different under different drought stress intensities or between different species. For example, in *Arabidopsis* and rice, raffinose family oligosaccharides (RFOs) is the earliest and most over accumulated metabolite in response to drought (Pires et al., 2016; Todaka et al., 2017), but myo-inositol only increased in water deficit stress and decreased in dehydration stress (Urano et al., 2009). The expression level of isocitrate lyase and malate synthase genes in rice (*Oryza sativa*) are closely correlated with the increase in glucose levels under drought stress, but there is no such correlation in *Arabidopsis*, it is suggested that glyoxylate cycle is involved in glucose accumulation under drought stress in rice, but not in *Arabidopsis* (Jin et al., 2017). Flavonoids often exist in the stable forms of *C*-glycosylation and *O*-glycosylation. Several uridine diphosphate (UDP)-dependent glycosyltransferase (UGT) have been identified in rice (*Oryza sativa*) (Brazier-Hicks et al., 2009), maize (*Zea mays*) (Falcone Ferreyra et al., 2013) and barley (*Hordeum vulgare*) (Zeng et al., 2020) to promote the synthesis of glycosylflavonoids. Among them, only the *C*-glucosyl transferase (*CGT*) in barley uses UDP-pentose instead of UDP-glucose as sugar base donor. These results provide the foundation for the systematic exploration of drought related metabolites and their regulatory genes in qingke.

To explore the metabolic mechanism and genetic basis of drought resistance in qingke, here, we conducted the evaluation of drought resistance, resequencing and analysing the metabolic profiling before and after drought stress on 246 qingke varieties. GWAS for drought-sensitivity index found that a root-specific expressed *CYP84* gene was significantly associated with drought resistance of qingke. In addition, although drought stress induced significant changes in some amino acids, flavonoids, saccharides and alcohols in qingke, the glycosylflavonoids with constitutive differences and not induced by drought stress are the key metabolites contributing to drought resistance of qingke. HORVU2Hr1G001460 is a glycosyltransferase gene necessary for the biosynthesis of *C*-glycosylflavonoids and contributing to drought resistance in qingke. These findings increase our knowledge of metabolites during drought stress and provided theoretical basis for drought resistance breeding.

## Materials and methods

### Plant materials

The 246 qingke varieties used in this study were taken from a collection in the State Key Laboratory of Hulless Barley and Yak Germplasm Resources and Genetic Improvement, including 44 cultivars, 181 landraces and 21 semi-wild varieties (**Supplemental Table 1**), of which semi-wild qingke does not exists as wild populations, but as weeds at the edges of field (Dequan, 1988). All qingkes were grown in the pots in a greenhouse with 22°C/18°C (day/night), 16/8h (light/dark) and 50% humidity. For treatment group, 10DAG (days after germination) seedlings were watered with 20% PEG 6000 instead of water to simulate drought stress. Each variety is planted with three replicates, with at least 15 plants in each replicate. Leaf wilting represents leaf dehydrating. The complete plant wilting is following by rolling, yellowing and scorching of leaves. Here, if the leaf is rolling or yellowing, it is a wilted leaf. After drought treatment, we count the number of wilted leaves and divide it by the total number of leaves to calculate the wilting rate. For example, the total number of plants germinated in a single replicate is 15, each plant has 3 leaves, the wilting rate after drought treatment is 0, which means that the leaves of all plants are not rolled or yellowed; 1%~25% wilting rate indicates that the number of rolled or yellowed leaves is between 1 and 11 (15*3*25%=11.25). During the drought treatment, the wilting rate of different repetitions of all varieties were manually counted every 24 hours from 72 hours to 120 hours, unless the germination rate was too low to result in less than 15 seedlings (Not applicable, NA). We divided these qingkes into five drought-resistance grades according to the wilting rate after drought stress, which were 0 (wilting rate of 0), 1 (wilting rate of 1% to 25%), 2 (wilting rate of 26% to 50%), 3 (wilting rate of 51% to 75%) and 4 (wilting rate of 76% to 100%). Grade 0 is the most drought resistant grade and grade 4 is the most sensitive grade (**Supplemental Table 1**). Drought-sensitivity index (DSI) calculated based on drought-resistance grades was used for drought resistance assessment. The calculation of DSI is according to the formula:


$$DSI=\frac{Grade_{72h}+Grade_{96h}+Grade_{120h}}{3}$$

The leaves of control group and the treatment group were taken from three different plants per line. Seedling leaves treated for 48 hours with same conditions (24 hours before the first appearance of wilting) were used for metabolites profiling. All samples were harvested at 9:00-11:00 on that day, placed in liquid nitrogen and stored at -80 °C immediately until vacuum freeze-drying.

### Genome resequencing and SNP calling

DNA from 246 qingke seedling leaves was exracted for whole genome resequencing, and sequencing libraries with short inserts were constructed following manufacturer’s in structions. Samples were sequenced on an Illumina HiSeq X ten platform. To retain reads of high quality, reads with fewer than 5% N (missing) bases and with fewer than 50% of bases of base quality< 5 were deemed as cleaned reads by fastp (version 0.19.3). All other reads were discarded. The sequence data have been deposited in NCBI Sequence Read Archive (SRA) database (**Supplemental Table 1**, PRJNA675977, https://www.ncbi.nlm.nih.gov/bioproject/?term=PRJNA675977). Then clean reads were mapped to barley IBSC_v2 reference genome (Mascher et al., 2017) with BWA (version 0.7.8-r455, mem -t 4 -k 32 -M) and further filtered by samtools (version 0.1.19). Finally, after filtration with minor allele frequency (MAF) greater than 0.05 and missing call frequency (MCF) less than 0.1, a total of 7,744,571 SNPs among 246 qingke accessions were identified by samtools (mpileup -m 2 -F 0.002 -d 1000) and bcftools (version 1.7) (Li, 2011), SNPs were further annotated by ANNOVAR (version 2013 Aug23). The genetic variants data have been deposited in European Variation Archive (EVA, https://www.ebi.ac.uk/eva/) database with accession number PRJEB57548.

### Metabolite profiling

Approximately 70mg of lyophilized leaves of each sample were crushed and extracted overnight at 4 °C with 1.0 mL 70% aqueous methanol before analysis using an LC-ESI-MS/MS system (HPLC, Shim-pack UFLC SHIMADZU CBM30A system; MS, Applied Biosystems 6500 plus Q TRAP). The qualification of metabolites was performed according to a developed widely targeted metabolome method (Chen et al., 2013). The HPLC used a Waters ACQUITY UPLC HSS T3 C18 column (1.8 µm, 2.1 mm × 100 mm) at 40 °C with a solvent system of water (0.04% acetic acid): acetonitrile (0.04% acetic acid) following the gradient program of 95:5 V/V at 0 min, 5:95 V/V at 11.0 min, 5:95 V/V at 12.0 min, 95:5 V/V at 12.1 min, 95:5 V/V at 15.0 min, with a flow rate of 0.35 mL/min and injection volume of 2.0 μL. The ESI source operation parameters were used as following: ion source, turbo spray; source temperature 500 °C; ion spray voltage (IS) (+) 5500 V and (-) 4500V; ion source gas I (GSI), gas II (GSII), curtain gas (CUR) were set at 55, 60, and 35.0 psi, respectively; the collision gas (CAD) was medium. Instrument tuning and mass calibration were performed with 10 and 100 μmol/L polypropylene glycol solutions in Linear ion trap (LIT) and triple quadrupole (QQQ) modes, respectively. QQQ scans were acquired as MRM experiments with collision gas (nitrogen) set to 5 psi. DP and CE for individual MRM transitions was done with further DP and CE optimization. A specific set of MRM transitions were monitored for each period according to the metabolites eluted within this period.

Metabolite identification was conducted by match of mass spectrum to reference library MetWare database (MWDB). MWDB was constructed based on the standard compounds or public database like METLIN (Tautenhahn et al., 2012), some of the metabolites without standard secondary spectra are inferred by experience. Qualitative parameters contain accurate mass of metabolites, MS2 fragments, MS2 fragments isotope distribution and retention time (RT). The secondary spectrum and RT of the metabolites in the project samples are compared with MWDB, specifically, the MS tolerance and MS2 tolerance are set to 20 ppm, RT offset does not exceed 0.2 minutes.

### Genome-wide association analysis

We selected biallelic SNPs with minor allele frequency (MAF) ≥ 0.05 and missing call frequency (MCF) ≤ 0.1 for mGWAS using a linear mixed model (LMM) provided by GEMMA program with parameters “-maf 0.05 -hwe 0.0” (Zhou and Stephens, 2012). LMM can be represented as: *Y* = *SNP* + *Q + Kinship*+ *e*, where Y is a matrix of DSI or log2 transformed metabolite data (phenotype), SNP represents the matrix of markers, Q represents the population structure as the fixed effect, Kinship represents the kinship matrix between the individuals as random effect, and e is the residual. Population structure analyses were performed by ADMIXTURE software version 1.3.0 (Alexander and Lange, 2011) with parameters “–cv=10 –method block –acceleration qn3 -C 0.0001”, a total of 1174039 LD-pruned SNP set obtained through plink (version 1.9) (Purcell et al., 2007) with parameters “–indep-pairwise 50 5 0.2” used to analyze population structure and calculate K. GWAS analysis obtains the association with each marker (SNP) and phenotype *P*-value, the significant threshold of mGWAS after correction by SNP number is *P*-value ≤ 4.26E-08, thus to locate the genomic regions significantly associated with the metabolic content. Then we used R (www.r-project.org) to draw Manhattan graph in order to visually show the significant associated area and model effect. The adjacent significant SNPs with distance less than 1Mb were connected together as a significant locus, if this locus exceeds 30Mb, this locus will be abandoned, and then the most significant SNP with lowest P-value in this locus was defined as the lead SNP. To reduce the interference of false-positive SNP on the recognition of a significantly associated locus, the number of SNPs in each significant locus should be more than five.

### Statistical analysis

Principal component analysis and neighbor-jointing phylogenetic tree was performed with log2 transformed metabolite data to improve the normality. For hierarchical clustering analysis (HCA) in the study of developmentally-controlled accumulation and natural variation of metabolites, metabolite data were normalized by a min-max normalization. Identification of differential accumulation of metabolites were determined by partial least squares-discriminate analysis (PLS-DA) (Thévenot et al., 2015) with VIP (Variable Importance for the Projection) > 1.0, fold-change (≥1.5 or ≤0.67) and adjusted Student’s t-test *P-*value<0.05. PCA, HCA, PLS-DA and Student’s t-test were performed using R (www.r-project.org/) software and the packages they depend (ropls, version 1.16.0). To identify qingke genomic regions affected by domestication and improvement, we measured the level of genetic diversity (π) using a 1Mb windows for qingke populations using the VCFtools (version 0.1.16) software.

### Phylogenetic analysis

The amino acid sequences of reported genes were obtained from NCBI according to their accession numbers (http://www.ncbi.nlm.nih.gov/) (**Supplemental Table 2**). Candidate gene information in this study was obtained from the barley IBSC_v2 genome annotation. The alignment of amino acid sequences was performed using MUSCLE bundled in MEGA X, and neighbor-joining trees were constructed using MEGA X software with all default parameters (Kumar et al., 2018). The reliability of the reconstructed tree was evaluated using a bootstrap test with 1000 replicates.

### qRT-PCR analysis

Total RNA was extracted from selected samples using RNA extraction kit. The qRT-PCR was performed on a BioRed IQ5 Fast Real-Time PCR System (BIORED Ltd.) using the SuperReal PreMix Plus (SYBR Green) kit (Tiangen Biotech Co., Ltd., Beijing, China) according to the manufacturer’s instructions with the following process: 95°C for 5 min for predegeneration, then 40 cycles of 94°C for 30 s, 56°C for 30 s, and 72°C for 90 s. Each experiment was performed in triplicate for biological and technical repeats, and normalized using ADP-ribosylation factor (*HvADP-RF*) gene as internal control. Primers sequences for qRT-PCR are listed in **Supplemental Table 3**.

### Transient expression of heterologous protein in *Nicotiana benthamiana*

The full-length coding sequences of HORVU2Hr1G001460 was amplified and cloned into the plant binary vector pCAMBIA1300. The pCAMBIA1300-35S:GFP construct was used as the negative control. The above vectors were separately introduced into *A. tumefaciens* strain GV3101 to carry out infiltration of *N. benthamiana* leaves. After infiltration, the plants were first incubated in darkness for 12h and then transferred to blue light for 2 days. The Agrobacterium-mediated infiltration of *N. benthamiana* leaves was performed, as previously reported (Chen et al., 2011). These experiments data were from three independent biological samples.

## Results

### Drought resistance analysis at seedling stage of qingke

**Figure 1:**
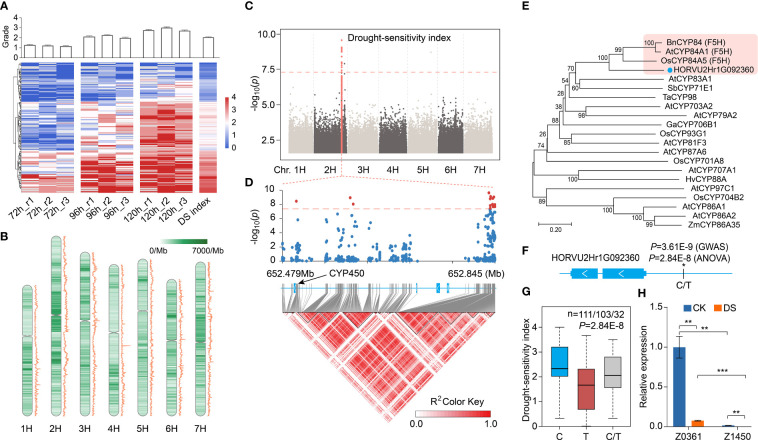
Drought resistance grading and functional variation of significant association sites of DSI-based GWAS. **(A)** The heat map shows the drought resistance grades of 246 qingke under drought stress for 72 hours, 96 hours and 120 hours (three biological replicates in each group). Blue indicates low wilting rate, and red indicates high wilting rate. The average grades of each variety in all groups are calculated as drought-sensitivity index (DSI). The upper bar graph shows the average grades of each group. **(B)** SNP distribution (heat map) and π value (line chart) along chromosome per 1Mb. **(C)** Manhattan plot displaying the GWAS result for the DSI. Orange dots indicate SNP in significance association loci. Gene model of *HORVU2Hr1G092360*, which is located 333 kb from the lead SNP (2H: 652834792), is shown. **(D)** A representation of the genes and pairwise R^2^ value among polymorphic sites in significant associated loci of DSI, where the darkness of the color of each box corresponds to the R^2^ value according to the legend. The scatter plot above represents the *P*-value of GWAS of each SNP in this area, red and blue indicate significant and non-significant SNPS, respectively. **(E)** An unrooted phylogenetic tree of CYP450 genes was constructed as described in Methods. Bootstrap values (based on 1000 replications) are indicated at each node (bar: 0.2 amino acid substitutions per site). **(F)** Gene model of HORVU2Hr1G092360, a filled blue box represents the exon. The “*” represents the SNP 2H:652503665 upstream of the gene, and the P-value in GWAS and ANOVA analysis of this SNP is also marked. **(G)** Boxplot showing the correlation between 2H:652503665 upstream of *HORVU2Hr1G092360* and DSI. C, T and C/T represent reference, alternate and heterozygous base. **(H)** Relative expression of *HORVU2Hr1G092360* in drought-resistant qingke (Z0361) and drought-sensitive qingke (Z1450) under CK and DS conditions, *HvADP-RF* gene as internal control. Data are shown as the mean ± SEM, n = 3. Student’s t-test, *P*-value< 0.01, marked with “**”. *P*-value< 0.001, marked with “***”.

246 accessions of qingkes collected in this study were treated with drought stress at the seedling stage, and we found that leaf wilting appeared for the first time after 72 hours of drought treatment. Wilting is the external manifestation of water potential in plants. It has been proved that leaf wilting is closely related to the plant drought resistance and can be an indicator for the evaluation of plant drought resistance (Alidu et al., 2019; Ye et al., 2020), scores based on the leaf wilting were also used for genome-wide association analysis in biotic (Paulino et al., 2021) or abiotic (Ahmed et al., 2021) stress research. To assess the drought resistance of highland barley directly and accurately, the wilting rate of qingkes under drought stress was counted and divided into 0 to 4 grades. 56.9% (140/246) of qingke varieties had a wilting rate lower than 25% (0 or 1 grade) after 72 h drought stress, and that about 66.7% (164/246) qingke varieties had a wilting rate of more than 50% (3 or 4 grade) after 120h drought stress (**Figure 1A**, **Supplemental Table 1**).

Average value of drought resistance grade after 72 h, 96 h and 120 h drought stress was calculated as drought-sensitivity index (DSI). DSI showed a continuous distribution, with the coefficients of variation (CV) and mean value of 50% and 2.05, respectively. There are 47 qingke varieties with a DSI value lower than 1, indicating stronger drought resistance, and 80 with a DSI value greater than 2.5, indicating weak drought resistance. Considering the consistency of biomass and wilting rate between repetitions, 20 extreme drought-resistant (R) and 20 extreme drought-sensitive (S) qingke varieties were finally determined, and the other 206 varieties were divided into intermediate types (**Supplemental Table 1**). As an indicator of membrane damage, especially during drought, the more severe damage due to drought, the higher the malondialdehyde content in plants (Laxa et al., 2019; Zhou et al., 2022).We further detected the malondialdehyde content in the leaves of ZYM2198 and Z1450, it was found that concentration of malondialdehyde in drought resistant variety (ZYM2198) was significantly lower than drought sensitive variety (Z1450) after 120h drought stress (**Supplemental Figure 1**).

### Genome-wide association studies based on drought-sensitivity index

A total of 10.44 terabases resequencing data were generated to analyze genomic variation of all varieties, and clean reads were mapped to the IBSC_v2 reference genome (Mascher et al., 2017) that is well assembled and annotated (**Supplemental Table 1**). After SNP calling, we selected SNP with missing call frequency of less than 10% and minor allele frequency more than 5%, resulting in a total of 7,744,571 SNPs, ranging from 893,125 (chr1H) to 1,508,037 (chr2H) on each chromosome, and the nucleotide diversity π (windows 1Mb) was in the range of 2.2e-07 to 2.0e-03 (**Figure 1B**, **Supplemental Table 4**). The best fitting model of admixture indicated by the minimum of cross validation error was K=6 in population structure analysis, with a clear pattern of subdivision among the cultivar and semi-wild (**Supplemental Figure 2**).

We then performed GWAS of DSI in 246 qingke lines by a linear mixed model (LMM) to identify the associated genome loci and drought resistance related genes, the genome-wide significance threshold (*P*_LMM_) was set to 4.26e-08 after correction by the number of effective-independent SNPs (Li et al., 2012) (**Supplemental Table 5**). A total of 17 SNPs were significantly associated with the DSI, and 15 SNPs from 652.5Mb to 652.8Mb on chr2H showed strong peak, among which the lowest *P*-value of SNP (lead SNP) in this locus is 2.38E-10 (2H:652834792) (**Figure 1C**). Five genes are located on this region, including a cytochrome P450 family gene (*CYP450*, HORVU2Hr1G092360), a Kinesin like protein (HORVU2Hr1G092370), a p-loop NTPase domain containing protein (HORVU2Hr1G092390) and two jumonji-like transcription factor gene (HORVU2Hr1G092380, HORVU2Hr1G092400). Except for SNPs detected in the promoter of *CYP450* gene, the other four genes had no genomic variation in the gene body or its promoter (**Figures 1C, D**). *CYP450* family genes have been reported to participate in plant stress resistance through the biosynthesis of secondary metabolites and phytohormones (Tian et al., 2004; Mao et al., 2013; Pandian et al., 2020). In the phylogenetic tree of *HORVU2Hr1G092360* and CYP genes with clear function reported, *HORVU2Hr1G092360* showed a high bootstrap value to the *CYP84* gene that function as ferulate-5-hydroxylase (*F5H*) from *Arabidopsis thaliana* and *Oryza sativa* (**Figure 1E**). *F5H* is a key gene of lignin synthesis pathway in plants, and genetic evidence suggests that *CYP84* gene in rice is involved in ultraviolet tolerance (Ruegger et al., 1999; SASAKI et al., 2010).

By One-way analysis of variance (ANOVA), it was found that SNP 2H:652503665 (C/T) with GWAS significance (*P*=3.61E-9) at 835bp upstream of the transcription start site of *HORVU2Hr1G092360* showed compelling difference (*P*=2.84E-8) in DSI (**Figure 1F**), further Fisher’s least significant difference (LSD) procedure showed that DSI between any two genotypes was significantly different (*P*-value, C vs T: 3.9E-09, C vs C/T: 0.035, T vs C/T: 0.043). Average DSI of qingkes with SNP 2H:652503665 as C was 2.44, as T was 1.64, and DSI in heterozygous is between them (**Figure 1G**). Besides, in about 80% of drought-resistant qingkes, this site was a T, while C in 90% of drought-sensitive qingkes, with the remainder being heterozygous. In the intermediate qingkes, the proportion of two alleles was similar (46.1% vs 39.8%) (**Supplemental Figure 3**). Moreover, by searching the expression database of barley gene (Zhou et al., 2022b), it was found that *HORVU2Hr1G092360* was primarily expressed in root, which was consistent with its homologous gene in tomato (Vasav and Barvkar, 2019). qRT-PCR showed that the expression of this gene in the root of drought resistant qingke (Z0361, T) was significantly higher (Student’s t-test *P*-value, CK: 2.0E-3, DS: 2.2E-4) than that of drought sensitive qingke (Z1450, C), and the expression was down regulated by drought stress (Student’s t-test *P*-value, Z0361: 2.5E-3, Z1450:2.8E-3) (**Figure 1H**). Therefore, we speculated that *HORVU2Hr1G092360* is involved in drought tolerance regulation in qingke through lignin synthesis pathway.

### Metabolic profiling of qingke

**Figure 2:**
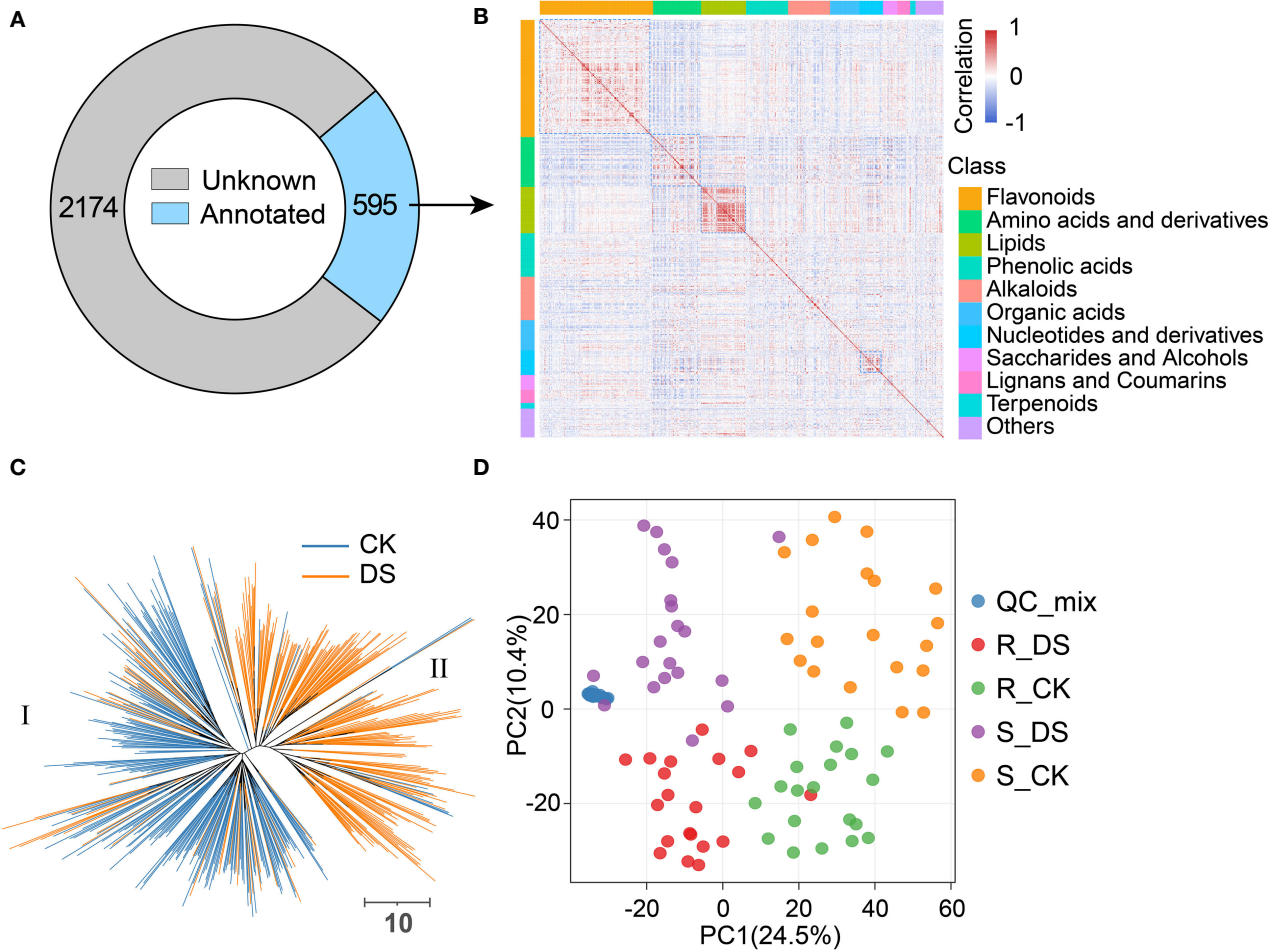
Classification and statistics of metabolites in qingke. **(A)** Metabolic features detected in qingke leaves, unknown and annotated are represented in gray and light blue, respectively. **(B)** Heatmap of correlation (spearman) among annotated metabolites, red indicates positive correlation and blue indicates negative correlation. The color blocks on the top and left represent the class of metabolites. **(C)** Neighbor-jointing phylogenetic tree constructed using metabolites identified in qingke leaves before and after drought stress. Two major clades, represented by CK and DS, were identified from the tree. The scale bar indicates the simple matching distance. **(D)** Principal component analysis (PCA) score plot showing distinctive metabolic profiles of drought-resistant qinge (R) and drought-sensitive qingke before (CK) and after (DS) drought stress. The blue dot represents the mixed sample used for the quality control in metabolite detection.

In actual field condition, most of the cases are mild stresses that threaten the growth and development of plants. However, the response of plants to mild drought stress is poorly understood compared with severe water stress (Clauw et al., 2015). Leaves of plants grown in optimal condition (control check, CK) and subjected to 48h mild drought stress (DS) at the seedling stage were collected from 246 accessions of qingke, then, an MS2 spectral tag library (MS^2^T) was constructed based on the liquid chromatography-tandem mass spectrometry (LC-MS/MS)-based widely targeted metabolic profiling method (Chen et al., 2013). Of the 2,769 metabolic features detected, 595 were identified using authentic standards or putative annotation. The most common of these annotated metabolites are flavonoids (167), followed by amino acids and derivatives (71), lipids (66), phenolic acids (62) and alkaloids (62) (**Figures 2A, B**, **Supplemental Table 6**). 55.7% (1,541/2,769) and 53.9% (1,492/2,769) of metabolites in CK and DS group had CV greater than 0.5, with an average of 0.74 and 0.68 (Student’s t-test *P* = 1.9E-3), respectively, indicating that the statistical dispersion of metabolites decreased after mild drought stress (**Supplemental Figure 4A**).

It is generally believed that the contents of chemically related metabolites are often interdependent. We found a large number of metabolite pairs with significant positive correlation by Spearman’s rank correlation, especially those in the same classification. There was a more remarkable positive correlation among lipids, flavonoids, amino acids and nucleotides than that of other metabolites (**Figure 2B**, **Supplemental Table 7**). For example, luteolin 6-*C*-glucosides and apigenin *C*-glucosides are both flavone *C-*glucosides with luteolin and apigenin as substrates and then modified by glycosylation, respectively. Similar substrates and enzymes lead to their close correlation in accumulation (r=0.85, *P<*2.2E-16). There are some metabolites such as indole-3-carboxaldehyde and 3-indoleacetonitrile that show a significant negative correlation (r=-0.52, *P<*2.2E-16), which may be due to their competition on indole (**Supplemental Tables 6**, **7**). We also observed a negative correlation between metabolites without similar chemical structure, such as inositol and amino acids, which may be due to their distinct patterns in response to drought stress (**Supplemental Tables 6**, **7**). In order to describe heterogeneity between samples from metabolome, we performed phylogenetic tree and principal component analysis (PCA) based on metabolite accumulation. The phylogenetic tree explicitly divided all samples into two clusters, which contain most of the CK (clade I) or DS (clade II) group, respectively (**Figure 2C**), and PCA showed similar results (**Supplemental Figure 4B**). This indicated that the metabolomics of qingke had a general change in response to mild drought stress. PCA also proved the difference between drought-resistant and drought-sensitive qingkes, whether before or after mild drought stress (**Figure 2D**).

### Metabolic changes in response to mild drought stress in qingke

**Figure 3:**
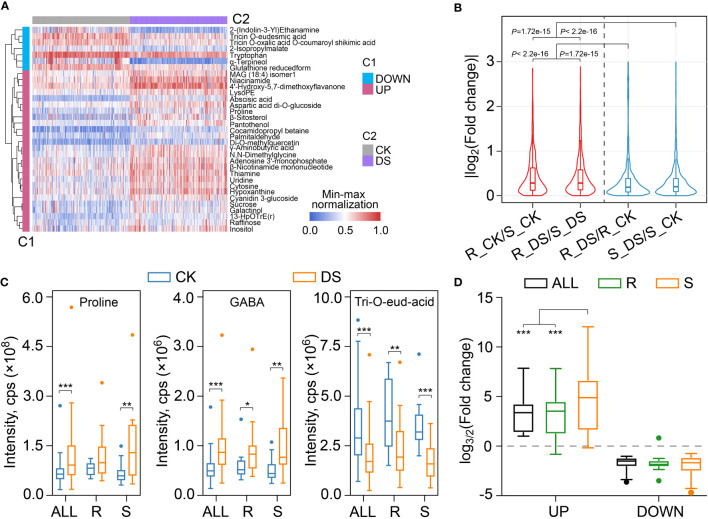
Metabolites in response to drought stress in qingke. **(A)** Heatmap visualization showing the metabolites accumulated differentially based on all qingke population in response to drought stress. The contents of each metabolite were normalized before performing linkage hierarchical clustering. Blue indicates the down regulated metabolites after drought stress, and red indicates the up-regulated metabolites after drought stress. **(B)** Violin plot showing the fold change differences between drought-resistant qingkes before and after mild drought stress (R_ DS/R_ CK), drought-sensitive qingkes before and after mild drought stress (S_ DS/S_ CK), the drought-resistant and drought-sensitive qingkes in CK condition (R_CK/S_ CK), drought-resistant and drought-sensitive qingkes after mild drought stress (R_DS/S_ DS). Red indicates the fold change of metabolites between drought-resistant and drought-sensitive qingkes under the same conditions, and blue indicates the fold change of metabolites of drought-resistant or drought-sensitive qingkes in response to drought stress. Wilcox-test P-value is also displayed. **(C)** The content of proline, GABA and Tricin *O*-eudesmic acid in all qingkes (ALL) populations, drought-resistant qingkes (R) and drought-sensitive qingkes (S) before (CK) and after (DS) drought stress. Student’s t-test, P-value < 0.05, marked with “*” *P*-value < 0.01, marked with “**” *P*-value< 0.001, marked with “***”. **(D)** The box plot shows the fold change of up-regulated and down-regulated metabolites based on all qingke populations in response to drought stress in all qingke populations (ALL, black), drought-resistant (R, green) and drought-sensitive (S, orange) qingke.

In order to provide insights into the universal metabolic response under mild drought stress, we analyzed the differentially accumulated metabolites of all qingkes between the CK and DS group (**Supplemental Figure 5A**) by VIP (Variable Importance for the Projection) > 1.0, fold-change (≥1.5 or ≤0.67) and adjusted Student’s t-test *P-*value< 0.05. A total of 268 significantly up-regulated and 34 down-regulated metabolic features were identified after 48 h mild drought stress, of which 26 and 7 were annotated, respectively (**Supplemental Table 8**). Among the up-regulated metabolites, nucleotides and saccharides are the most abundant ones with 5 each, while the fold changes of abscisic acid and pantothenol are the largest, with an average increase of 5.53 and 3.38 times, respectively (**Figure 3A**). The down-regulated metabolites mainly included flavonoids, amino acids and the most significant down-regulation, α-terpineol, which was reduced by 3.19 times after mild drought stress.

We also compared the changing intensity of metabolites between different types of qingke during mild drought stress. Both drought-resistant and drought-sensitive qingkes had subtler changes in metabolites in response to mild drought stress than their constitutive differences under the same conditions (Wilcox-test *P*-value<0.001) (**Figure 3B**). This hypothesis is also verified by comparing the maximum and median of their fold change. The maximum fold change of metabolites accumulation of R_CK vs S_CK and R_DS vs S_DS can reach 119.7 and 115.8, respectively, while that of R_DS vs R_CK and S_DS vs S_CK are only 22.3 and 77.4, respectively. The median values of these four comparison groups are 1.22, 1.22, 1.14 and 1.16 (**Supplemental Table 9**). In short, the difference of constitutive metabolites between contrasting drought-resistant types of qingke is greater than that of inducible metabolites after 48 h mild drought stress before wilting.

Some reported marker metabolites of abiotic stress were also over accumulated after mild drought stress in qingke, in which proline increased by an average of 1.71 times, 4-aminobutyric acid (GABA) increased by an average of 1.74 times. In addition, although cyanidin 3-glucoside and di-*O*-methylquercetin with antioxidant and radical scavenging were increased after mild drought treatment, some other flavonoids such as tricin *O*-eudesmic acid and tricin *O*-oxalic acid *O*-coumaroyl shikimic acid were decreased (**Figure 3C**, **Supplemental Table 9**). It is worth noting that, the accumulation of proline and GABA in drought-sensitive qingkes are both lower than drought-resistant qingkes in CK, but higher than drought-resistant qingke after mild drought stress on average (**Supplemental Table 9**). We further analyzed fold changes of the above 268 increased and 34 decreased metabolites in different groups of qingke (All, R, S). The results showed that drought-sensitive qingkes have a larger fold change in these metabolites than ALL or drought-resistant qingkes. For the metabolites that were decreased after mild drought stress, there was no significant difference in their decreasing folds among different groups (**Figure 3D**). Compared with the drought-resistant qingkes, the drought-sensitive qingkes had more metabolites that specifcally up-regulated after drought stress, which also indicated the sensitivity of its metabolome in response to mild drought stress (**Supplemental Figure 5B**). Therefore, although the drought induced metabolites such as proline and GABA are increased after drought stress in both drought-resistant and drought-sensitive qingke, drought-resistant qingke is qualified to respond to drought stress more mildly due to its higher intrinsic related metabolism content, which may be contributed by other non-inducible metabolites under CK, these non-acutely increased metabolites are conducive for plants to adapt to stress environment.

### Genetic basis of metabolic response in qingke during drought stress

**Figure 4:**
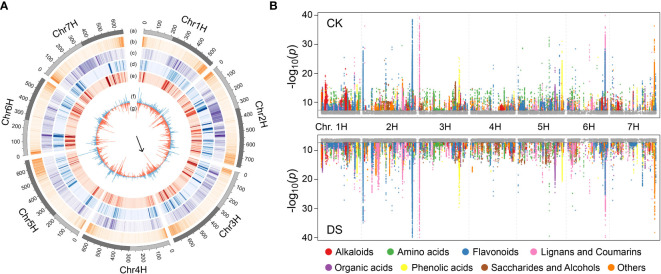
mGWAS of qingke under CK or DS condition. **(A)** Chromosomes distribution of genomic features involved in mGWAS. Lane a: long (dark grey) and short (light grey) arm of chromosome; Lane b: density (per Mb) of annotated genes; Lane c: density (per Mb) of SNP; Lane d: density (per Mb) of significantly associated loci in mGWAS under CK; Lane e: density (per Mb) of significantly associated loci in mGWAS under DS; Lane f: histogram (per 5Mb) of lead SNP in mGWAS under CK; Lane g: histogram (per 5Mb) of lead SNP in mGWAS under DS. The arrow highlights the difference in lead SNP density between CK and DS group at chr3H:485-490Mb. **(B)** Manhattan plots of mGWAS showing genetic associations for the annotated metabolites under CK or DS condition. The strength of association for the metabolites is indicated as the negative logarithm of the *P-*value for the LMM model. All metabolite-SNP in significantly associated loci were plotted in color.

In order to explore the genetic basis of metabolite response during drought stress, we performed mGWAS of qingke population under CK or DS condition, respectively. Estimation of broad-sense heritability (*H^2^*) showed that 58.7% (1,626 of 2,769) of the metabolites have a value greater than 0.5, with an average of 0.54 (**Supplemental Table 10**). A total of 266,440 significant SNPs for 1,006 metabolites were identified in qingke under CK, including 6,162 lead SNPs from 6,162 loci. In DS group, we detected 258,318 significant SNPs from 1,031 metabolites, including 7,618 lead SNPs from 7,618 loci (**Figure 4A**, **Supplemental Tables 11**, **12**). At least one significant locus was detected in these metabolites, the average phenotypic variance explained (PVE) of these significant SNPs are 15.0% and 15.6%, respectively. We then analyzed the distribution of significant SNPs across the chromosomes. Not surprisingly, the distribution of significant SNPs was independent to the gene density (Pearson correlation coefficient, CK: -0.005, DS: 0.004), but significantly correlated with the genomic SNP density (Pearson correlation coefficient, CK: 0.229, DS: 0.228, both *P-*value<2.2E-16) (**Figure 4A**). For lead SNPs, although they have comparable distribution under CK and DS across most of genomic regions, there are exceptions. For example, there were 76 lead SNPs in chr3H:485-490Mb under DS condition, while only 16 lead SNPs were detected in this region under CK condition (**Figure 4A**, indicated by the arrow). Therefore, the difference of metabolites accumulation in various environments affects genome-wide association studies in qingke, as well as the search of hotspot regions.

In addition, 3 (4-Pyridoxic acid, geniposidic acid, 3-3,4-Dihydroxyphenyl-2-methylalanine) and 1 (3-3,4-Dihydroxyphenyl-2-methylalanine) annotated metabolites colocalized with DSI under CK and DS conditions, respectively (**Supplemental Tables 11**, **12**). However, there was no obvious accumulation difference of these metabolites between drought-resistant qingke and drought-sensitive qingke (**Supplemental Table 8**), and the candidate gene *CYP84* identified above are expressed specifically in roots. Although colocalization may be just a coincidence, it provides ideas for us to find phenotype related metabolic pathways and candidate genes. 209 and 207 annotated metabolites had significantly associated loci in CK and DS, respectively, of which 142 were common, predominantly including flavonoids (68), alkaloids (19) and phenolic acids (15) (**Figure 4B**). Among the 65 metabolites that only identified significant loci in DS, amino acids and flavonoids were the most, with 19 and 12 respectively. It also includes two up-regulated metabolites galactinol and sucrose after mild drought stress, and two metabolites tricin O-oxalic acid O-coumaroyl shikimic acid and 2-(Indolin-3-Yl)Ethanamine down-regulated after mild drought stress. However, we cannot directly simply identify their regulatory genes through gene annotation in the significant loci (**Figure 3A**, **Supplemental Tables 11** and **12**), which may be due to the complex regulation involved in transcription factors. Besides, 46 annotated metabolites had the same lead SNP under CK and DS condition, indicating that these lead SNPs likely to be closed to functional gene regulating metabolites (**Supplemental Table 13**). For example, the lead SNP (7H:111181667) of salicylic acid *O*-glucoside is located in the HORVU7Hr1G040740 gene, as a homologous gene in *Arabidopsis*, AT2G14610 (*AtPR1*) was reported to be a marker gene in response to salicylic acid accumulation induced by a variety of pathogens (Ahmad et al., 2011; Eshraghi et al., 2011). HORVU7Hr1G040760 gene adjacent to HORVU7Hr1G040740 is annotated as glycosyltransferase, which is highly homologous with salicylic acid glycosyltransferase gene (AT2G43840, blastp E-value: 9E-44) in Arabidopsis (George Thompson et al., 2017). Therefore, changes in abundance of multiple metabolites under abiotic stress may lead to variable mGWAS results, but at the same time, those conservative significant association sites can effectively help us find key regulatory genes.

### Regulation of alpha-galactosidase on raffinose in response to mild drought stress

**Figure 5:**
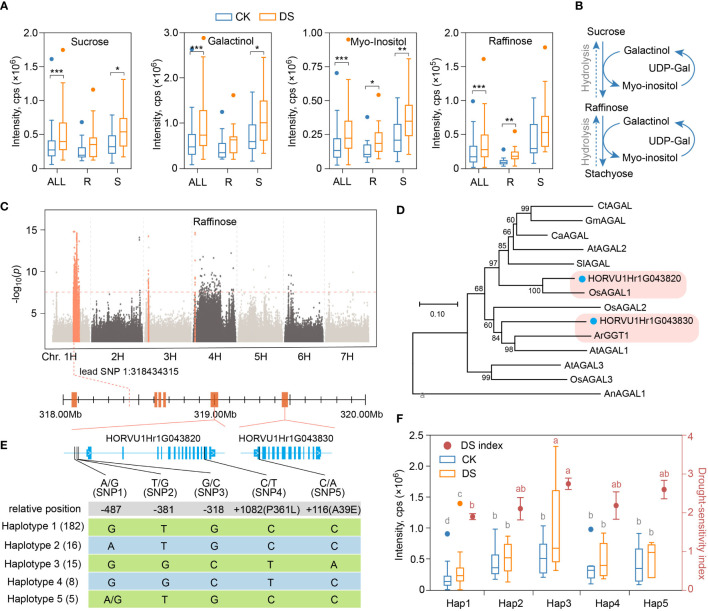
Alpha-galactosidase gene regulates raffinose synthesis induced by mild drought stress. **(A)** Boxplot showing the contents of sucrose, galactitol, myo-inositol and raffinose in all qingke accession (ALL), drought-resistant qingke **(R)** and drought-sensitive qingke (S) under CK (blue) and DS (orange) condition. **(B)** Schematic representation of the biosynthetic pathway of the raffinose. **(C)** Manhattan plot displaying the mGWAS results for the content of raffinose in CK condition. Orange dots indicate SNP in significance association loci, and two gene model of alpha-galactosidase found in the significance association loci is shown. **(D)** An unrooted phylogenetic tree of α-galactosidases was constructed as described in Methods. Bootstrap values (based on 1000 replications) are indicated at each node (bar: 0.1 amino acid substitutions per site). **(E)** The upper part is a representation of the genes in significant associated loci of raffinose from 1H:317.9Mb to 319.5Mb, and the lower part showed nucleotide polymorphisms identified near the HORVU1Hr1G043820 and HORVU1Hr1G043830, frequency of 5 haplotypes among these two genes in qingke population was labeled. **(F)** Raffinose content (boxplot, corresponding to the left y axis) and drought-sensitivity index (dot plot, corresponding to the right y axis) of qingke corresponding to the five alleles described in figure E under CK (blue) and DS (orange) condition, different letters on boxplot indicate significant differences according to LSD procedure after one-way ANOVA at *P*-value < 0.05, grey and red letters indicate metabolite content and DSI, respectively. Student’s t-test, P-value < 0.05, marked with “*” P-value < 0.01, marked with “**” P-value < 0.001, marked with “***”.

Among the 26 metabolites that accumulated significantly after mild drought stress, five were saccharides and alcohols, among which sucrose, galactinol, myo-inositol and raffinose were increased by 1.53, 1.61, 1.62 and 1.55 times, respectively (**Figure 5A**). Plants synthesize raffinose with galactinol as galactosyl donor and sucrose as receptor by raffinose synthase, and release myo-inositol at the same time. Raffinose can also be added with a galactosyl groups to form stachyose of another member of raffinose family oligosaccharides (RFOs) under the action of stachyose synthase, or hydrolyzed into sucrose and galactose under the hydrolysis action of α-galactosidase (**Figure 5B**). Surprisingly, not that the higher the content of sucrose, myo-inositol, galactinol and raffinose after drought stress, the lower wilting rate and higher drought resistance of qingkes. On the contrary, under both CK and DS condition, the contents of these metabolites in drought-sensitive group were higher than those in drought-resistant group (**Figure 5A**, **Supplemental Table 8**). Therefore, although raffinose and other saccharides or alcohols of its pathway showed overaccumulation in response to mild drought stress, and contribute to drought tolerance as cell osmoprotectants in plant, they were not the indicators or crucial metabolite for drought resistance evaluation in qingke.

As a marker metabolite of early response to drought in qingke, the accumulation of raffinose is always lower in drought-resistant group before and after stress. To explore the genetic basis of this disparity, we searched its regulatory genes near the significant loci according to mGWAS results in CK (**Figure 5C**) and DS (**Supplemental Figure 6**). In the significant loci of chr1H:317.9-319.5Mb, there are six annotated genes, including two adjacent α-galactosidase genes that 563kb away from the lead SNP (1H:318434315, *P*=1.45E-15), HORVU1Hr1G043820 (HvAGAL1) and HORVU1Hr1G043830 (HvAGAL2) (**Figure 5C**). Both genes have alpha galactosidase A (PF16499) and alpha galactosidase C-terminal beta sandwich domain (PF17801). Phylogenetic tree analysis showed that HORVU1Hr1G043820 was highly homologous with *OsAGAL1* gene in rice (**Figure 5D**). *OsAGAL1* could effectively hydrolyze galactose-containing oligosaccharides *in vitro*, and the substrate specificity for raffinose was the highest (Kim et al., 2002; Li et al., 2007). Another gene, HORVU1Hr1G043830, had high homology with *ArGGT1* gene reported in common bugle (*Ajuga reptans*) (**Figure 5D**). Studies have shown that the recombinant enzyme from *ArGGT1* has galactan galactosyltransferase activity and some hydrolytic (α-galactolytic) activity (Tapernoux-Lüthi et al., 2004). In addition, both genes have signal peptide cleavage sites, which was a conservative feature of this enzyme entering the lumen of the endoplasmic reticulum during its translation (Feurtado et al., 2001)(**Supplemental Figure 7**).

We then performed polymorphism analysis of SNP near *HvAGAL1* and *HvAGAL2* genes, including 3 upstream SNP and 2 non-synonymous SNPs (**Figure 5E**). Although neither of the two non-synonymous SNP was in the predicted conserved domain, the SNP5 resulted in a change in amino acids polarity (nonpolar to polar). These SNPs can be mainly combined into five haplotypes, and the 20 qingke varieties in drought-resistant group were all haplotype1, while only one variety in the drought-sensitive group were haplotype1 (**Supplemental Figure 8**). Raffinose accumulation corresponding to the five haplotypes was also significantly different. Specifically, in CK group, the raffinose content with haplotype3 was 2.43 times that of haplotype1, and in DS group, haplotype3 was 3.43 times that of haplotype1, while raffinose carring the other three haplotypes were between them with no significant difference. Not surprisingly, qingke varieties with haplotype1 had the lowest drought-sensitivity index, while varieties with haplotype3 had the highest drought-sensitivity index (**Figure 5F**). There were also significant differences in raffinose content between qingke varieties with haplotype1 and haplotype2, as well as between haplotype3 and haplotype4 in DS group (**Figure 5F**), suggesting that both *HvAGAL1* and *HvAGAL2* were involved in the regulation of drought resistance related raffinose metabolism in qingke.

### The constitutive *C*-glycosylflavonoids regulated by glycosyltransferase enhance drought resistance in qingke

**Figure 6:**
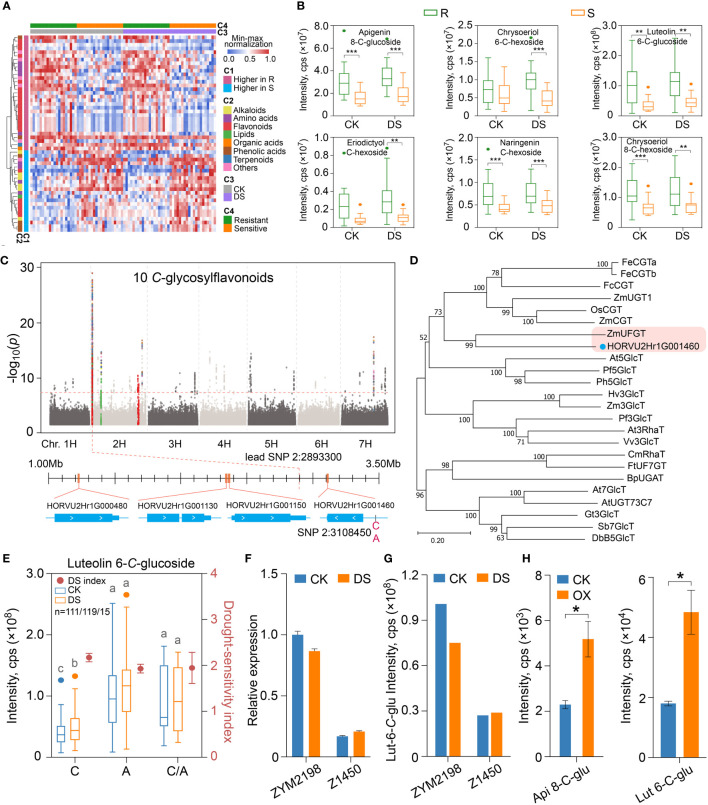
The synthesis of constitutive *C*-glycosylflavonoids that contribute to drought resistance is regulated by a *C*-glucosyl transferase gene in qingke. **(A)** The heatmap showing the metabolites accumulated differentially in drought-resistant and drought-sensitive qingke after drought stress, and the accumulation of these metabolites before and after drought stress. C1: differential patterns of metabolites; C2: classification of metabolites; C3: control (CK) or under drought stress (DS); C4: grouping of drought-resistant (R) and drought-sensitive (S) qingke. **(B)** The boxplot represents the *C*-glycosylflavonoids with enhanced accumulation in drought-resistant qingke (R, green) than that in drought-sensitive qingke (S, orange) under CK and CS condition. **(C)** Manhattan plot displaying the mGWAS co-location of 10 *C*-glycosylflavonoids in CK condition. Colored dots indicate SNPs in significance association loci. Four gene models representing CYP450 (HORVU2Hr1G000480, HORVU2Hr1G001130, HORVU2Hr1G001150) and glycosyl transferase (HORVU2Hr1G001460) found in the significance association loci are shown. **(D)** An unrooted phylogenetic tree of glycosyl transferase was constructed as described in Methods. Bootstrap values (based on 1000 replications) are indicated at each node (bar: 0.2 amino acid substitutions per site). **(E)** Luteolin-6-*C*-glucoside content (boxplot, corresponding to the left y axis) and drought-sensitivity index (dot plot, corresponding to the right y axis) of qingke corresponding to the SNP 2H:3108450 under CK (blue) and DS (orange) condition, C, A and C/A represent reference, alternate and heterozygous base, different letters on boxplot indicate significant differences according to Students’t test at *P*-value < 0.05. **(F)** Relative expression of HORVU2Hr1G001460 gene in drought-resistant qingke (ZYM2198) and drought-sensitive qingke (Z1450) under CK and DS conditions. Data are shown as the mean ± SEM, n = 3. **(G)** The contents of luteolin-6-*C*-glucoside in ZYM2198 and Z1450 under CK and DS conditions, respectively. **(H)** Accumulation of apigenin-8-*C*-glucoside (api 8-*C*-glu) and luteolin-6-*C*-glucoside (lut 6-*C*-glu) in tobacco leaves that transiently overexpress HORVU2Hr1G001460. The asterisk represents a significant difference of Students’t test at *P*-value < 0.05 between CK and overexpression line (OX). Data are shown as the mean ± SEM, n = 3. Student’s t-test, P-value < 0.05, marked with “*” P-value < 0.01, marked with “**” P-value < 0.001, marked with “***”.

Drought stress is a continuous aggravating process, in order to explore its metabolic basis, we focused on the metabolites accumulated differently between drought-resistant and drought-sensitive qingkes after 48 hours of mild drought stress. A total of 154 metabolites that accumulate higher in drought-resistant qingke were identified, most of which have been annotated were flavonoids (17/31, 55%), such as homoorientin (Luteolin-6-C-glucoside), vitexin (Apigenin 8-C-glucoside) and orientin (Luteolin 8-C-glucoside) (**Supplemental Table 9**). We also identified 153 metabolites with enhanced accumulation in drought-sensitive qingke, including 23 annotated metabolites such as hordenine, glutamic acid, narirutin and sinapic acid, as well as flavonoids and alkaloids (**Figure 6A**). Among 307 differential metabolites between R_DS and S_DS group, 95.4% (293/307) did not change significantly in the comparison before and after drought stress in ALL group (**Supplemental Table 8**). Besides, about 60% (181/307) of the differentially accumulated metabolites between R_DS and S_DS also had significant differences before their drought stress (**Supplemental Table 9**). Therefore, we believe that the differentiation of drought tolerance in qingke population is mainly caused by functional metabolites with constitutive differences.

As shown in **Figure 6A**, 54 annotated metabolites accumulated differently between R_DS and S_DS were not induced by mild drought stress, so that the disparity could be maintained. We then pay close attention to 10 flavonoids with similar accumulation patterns that higher in drought-resistant qingke and not inducible by drought (**Figure 6A**). All of them were modified with *C*-hexosylation (apigenin 8-*C*-glucoside, chrysoeriol 6-*C*-hexoside, luteolin 6-*C*-glucoside, luteolin 8-*C*-glucoside, luteolin *C*-hexoside, luteolin 6-*C*-hexoside, eriodictyol *C*-hexoside, apigenin *C*-glucoside, naringenin *C*-hexoside, chrysoeriol 8-*C*-hexoside), include 2 flavanones and 8 flavones (**Supplemental Table 9**). The accumulation of these ten flavonoids in drought-resistant qingke after mild drought stress is 2.32 times higher than that of drought-sensitive group on average, and the highest can reach 3.38 times (**Figure 6B**, **Supplemental Figure 9A**).

In addition to the similarity in chemical structure, these ten flavonoids were also co-localized on chr2H:1.2-3.3Mb in mGWAS under CK and DS condition, and the *P*-value of lead SNP (chr2H:2893300, apigenin-*C*-glucoside) was 9.84E-30 (**Figure 6C**, **Supplemental Figure 9B**). When searching for candidate genes, we found that a total of 68 genes were annotated in this genomic region. In order to screen candidate genes, we further focused on three *CYP450* family genes (HORVU2Hr1G000480, HORVU2Hr1G001130, HORVU2Hr1G001150) and one UDP-glucosyltransferase (*UGT*) gene (HORVU2Hr1G001460) by combining known flavonoid pathway genes, chemical modification characteristics of these flavonoids and gene function annotation (**Figure 6C**). SNPs in gene body and upstream 2Kb regions of these four genes were also significantly correlated with the *C*-glycosylflavonoids content by one-way ANOVA (**Supplemental Table 14**). However, phylogenetic analysis showed that these three CYP450 genes were not clustered with *CYP93* or *CYP75* genes that involved in flavonoid metabolism, but closer to *CYP76* or *CYP86* (**Supplemental Figure 9C**), which are related to the process of geraniol and fatty acid metabolism (Bak et al., 2011, 45). The other *UGT* gene is 212kb away from the lead SNP. On the other hand, the *UGT* gene is clustered with *CGT* gene family in phylogenetic tree, and had the highest homology with the *ZmUFGT* (GRMZM2G063550) gene in maize, which was reported to be a UDP-glycosyltransferase gene involved in the metabolism of apigenin di-*C*-hexoside (Wen et al., 2016) (**Figure 6D**).

Due to the assembly gap of HORVU2Hr1G001460 gene on IBSC_v2 reference genome, we found that this gene corresponds to the HORVU.MOREX.r3.2HG0096540 gene in latest IBSC_v3 version genome (Mascher et al., 2021) by BLAST (Altschul et al., 1990), and SNP 2H:3108450 located at 797bp upstream of HORVU.MOREX.r3.2HG0096540 was significantly associated with C-glycosylflavonoids (**Figures 6C, E**, **Supplemental Table 14**). For example, under optimal growth conditions, the average content of luteolin 6-*C*-glucoside in qingke with base A (n=119) was 2.43 times that of base C (n=111), and 2.37 times under mild drought stress. However, there was no significant difference in *C*-glycosylflavonoids content between the C/A and A in this site, and DSI corresponding to A is also significantly lower than that of C (*P*-value=0.034) (**Figure 6E**). Besides, in 70% of drought-resistant qingkes, the base of SNP 2H:3108450 is A, while most (65%) of drought-sensitive qingkes, there is C. In intermediate group of qingke, the frequency of base C and base A of this SNP is 45% and 49%, respectively.

We also analyzed the expression of HORVU2Hr1G001460 in qingke varieties by qRT-PCR. In both drought-resistant and drought-sensitive qingkes, there was no significant change in the expression of HORVU2Hr1G001460 before and after drought stress, but ZYM2198 (drought-resistant, A in 2H:3108450) was 5.9 times and 4.2 times higher than Z1450 (drought-sensitive, C in 2H:3108450) in the expression of HORVU2Hr1G001460, respectively (**Figure 6F**), which was similar to the accumulation trend of *C*-glycosylflavonoids such as luteolin 6-*C*-glucoside in ZYM2198 and Z1450 (**Figure 6G**). In order to confirm the positive regulation of HORVU2Hr1G001460 on *C*-glycosylflavonoids synthesis, *in vivo* function of HORVU2Hr1G001460 was also verified by heterologously expressing HORVU2Hr1G001460 in *N. benthamiana* leaves. Compared with the control group, the content of these ten *C*-glycosylflavonoids in tobacco leaves with overexpressing of HORVU2Hr1G001460 gene all increased, and there were significant differences in four of them (**Figure 6H**, **Supplemental Figure 10**). In summary, we fully believe that the HORVU2Hr1G001460 gene, which encodes *CGT*, is a key structural gene of drought resistance-related *C*-glycosylflavonoids metabolism in qingke, and the SNP located at the promoter may directly affect the expression of this gene, thus resulting in the differentiation of the accumulation of *C*-glycosylflavonoids involved in the drought tolerance. Therefore, this *CGT* gene and SNP marker are of great significance in drought resistant qingke or barley molecular breeding.

## Discussion

Many studies have focused on mechanisms how to cope with severe drought stress. However, plants must adapt to changing environmental parameters in field, and extreme long-term drought that threatens the survival of crops will rarely occur. Therefore, it is more common for crops to suffer from mild drought stress at different development stages, resulting in varying degrees of yield loss (Clauw et al., 2015). Studies in *Arabidopsis* showed that RFOs, glucose and fructose responded to drought stress earlier than other metabolites, among which raffinose in RFOs pathway responded the earliest, followed by galactinol and inositol (Fàbregas et al., 2018). Similar results were also found in rice (Todaka et al., 2017). In this study, not only sugars such as sucrose and raffinose, inositol and galactinol of RFOs pathway were significantly over accumulated under mild drought stress, but also significantly accumulated ABA, GABA and proline were detected. Unexpectedly, we found that tryptophan and glutathione in qingke decreased after mild drought stress (**Supplemental Table 8**). It can be seen that the metabolites in response to early mild drought stress in plants mainly include glucose, fructose, sucrose, raffinose, inositol, galactinol, proline, tryptophan, GABA and ABA. Although the number and response patterns of these metabolites are different in various plants, more researchers believe that the accumulation of sugars and RFOs under drought stress is earlier than that of amino acid and ABA (Fàbregas and Fernie, 2019). Compared with conservative metabolic response during mild drought stress, the metabolome response patterns of different species under severe stress are quite different, and even the trend of citrate and succinate are opposite in *Arabidopsis* and rice (Fàbregas and Fernie, 2019). This also shows the diversity of metabolic response when plants cope with severe drought stress that threatening their own survival, which may be related to the experimental conditions and methodological approach. Therefore, in future studies, more powerful conclusions may be obtained by unification of growth status, drought treatment methods and metabolite analysis among different species.

At the same time, the constitutive difference of metabolites caused by population genetic diversity are universal, such as the difference of metabolites accumulation related to stress resistance among extreme varieties. Raffinose and vanillic acid were constitutively different metabolites among different resistance of rice cultivars, and their content were also induced by drought and heat stress (Lawas et al., 2019). Moreover, our previous studies found that demonstrated co-selection of both constitutive and induced phenylpropanoids for UV-B protection in qingke (Zeng et al., 2020). Therefore, it is of great significance to study whether the simultaneous accumulation of constitutive and inducible functional metabolites endows qingke with stronger drought resistance, which requires needs a lot of analysis and verification experiments to fully characterize this response of qingke.

In our study, we found that among the 289 differentially accumulated metabolites between drought-resistant and drought-sensitive qingke under control conditions, only 20 were up-regulated or down-regulated after drought stress. Surprisingly, glutathione and α-terpineol with enhanced accumulation in drought-resistant qingke were down-regulated after drought stress, while raffinose with high content in drought-sensitive qingke was up-regulated after drought stress (**Supplemental Tables 8**, **9**). Most of the (62.6%, 181/289) different metabolites between drought-resistant and drought-sensitive qingke before drought stress still showed constitutively significant differences after stress, such as apigenin-*C*-glucoside, naringin-*C*-hexside, syringenin 3-*O*-hexside, *N*-Acetyl-l-leucine, *N*-Acetylmethionine and 5-oxoproline. Interestingly, after mild drought treatment, many up-regulated metabolites have higher fold change and content in drought-sensitive qingke (**Figures 3C, D**), such as raffinose (**Figure 5B**). Thus, we speculate that the drought resistant differentiation of qingke population is mainly caused by constitutively different-accumulated metabolites, which may have sufficient ability to scavenge ROS and maintain cellular osmotic pressure, and are rarely induced by drought stress. Moreover, the moderate response of metabolites generally induced by mild drought stress in drought-resistant qingke can ensure its physiological function, and excessive response may bring other unbearable damage.

Raffinose and its metabolic pathway are unique to plants. They accumulate under abiotic stress of low temperature, drought, high temperature and high salt (Krasensky and Jonak, 2012; ElSayed et al., 2014). Here, we not only identified two adjacent α-galactosidase gene (HORVU1Hr1G043820 and HORVU1Hr1G043830) through mGWAS and gene function analysis that may regulate raffinose content in qingke, but also determined the advantageous and disadvantageous combination of natural variation on these two genes. In addition, the homologous genes of these two genes in *Arabidopsis*, rice, maize, *Brachypodium distachyon* and wheat are also adjacent on the chromosome, indicating their evolutionary conservation (**Supplemental Table 15**). qRT-PCR showed that the response trends of HORVU1Hr1G043820 and HORVU1Hr1G043830 after mild drought treatment were not consistent. HORVU1Hr1G043820 was down-regulated by drought stress, while HORVU1Hr1G043830 was up-regulated by drought stress, indicating that their functions may be differentiated. Based on the enhanced expression of HORVU1Hr1G043830 and lower raffinose content in ZYM2198 (drought-resistant) no matter under CK or DS condition, we speculate that HORVU1Hr1G043830 encoding a α-galactosidase with raffinose as substrate, but it is unclear why the content of raffinose increased with the increase of HORVU1Hr1G043830 expression after mild drought stress, which may be regulated by other raffinose synthesis-related genes besides raffinose degradation pathway (**Supplemental Figure 11**).

Flavonoid glycosides, such as chrysoeriol glucoside, apigenin glucoside, luteolin glucoside and anthocyanins accumulate under abiotic stress. *C*-glycosylflavonoids are the major class of flavonoids in cereal crops, and *C*-glycosylation is considered to be the key step in the synthesis of *C*-glycosylflavonoids. We found that compared with drought-sensitive qingkes, the over accumulated metabolites in drought-resistant qingkes are mainly flavonoid glycosides, especially *C*-glycosylflavonoids whose biosynthesis were catalyzed by a UDP-glucosyltransferase for *C*-hexosylation (*CGT*). Moreover, we screened two ethyl methanesulfonate (EMS)-mutagenized barley lines in *CGT* gene (*CGT#4, CGT#6*), and they carried missense mutations at positions 437 (Arg to Trp) or 387 (Gly to Asp), respectively (Jiang et al., 2022). The two mutant lines showed higher wilting rates (WT: 60%, *CGT#4*: 80%, *CGT#6*: 64%) and water loss rates than wild type after 72 h of drought stress (**Supplemental Figure 12**). Because the randomness of EMS mutagenesis, mutated lines will carry a different set of mutations, it is necessary to evaluate the contribution of *CGT* gene to drought resistance through stable transformation or genetic backcross experiments. Unlike the drought stress-induced flavonoid glycosides previously found in qingke, such as luteolin 7-*O*-glucoside and its regulatory gene *O*-glucosyl transferase (*OGT*) (Xu et al., 2021), the content of *C*-glycosylflavonoids and expression of *CGT* was not increased or decreased significantly after mild drought treatment in this study (**Figure 6A**). Whether this is due to the insufficient intensity of drought stress or the specific metabolic mechanism of the *C*-glycosylflavonoids needs to be further explored, but in any case, we believe that the variation in the promoter region of *CGT* gene affects its expression, which is an important reason for the differentiation of *C*-glycosylflavonoids content and resistance to drought stress in qingke population.

## Data availability statement

The original contributions presented in the study arepublicly available. This data can be found here: EuropeanVariation Archive, PRJEB57548, NCBI, PRJNA675977.

## Author contributions

YW designed the research and supervised this study. HY, YW and XZ participated in the material preparation. KY, LW, WZ and BW carried out the metabolite annotation and analyses. KY and LW performed the data analysis. XZ, KY and BW discussed the results, and KY and LW wrote the manuscript. All authors contributed to the article and approved the submitted version.
