# Supplementary material for: Genetic architecture of inducible and constitutive metabolic profile related to drought resistance in qingke (Tibetan hulless barley)
Source: Front Plant Sci. 2022 Dec 6;13:1076000. doi: 10.3389/fpls.2022.1076000 (PMC9763626; doi:10.3389/fpls.2022.1076000)
Supplement: Supplementary file 16 [file DataSheet_1.pdf]

Supplemental Figure 1

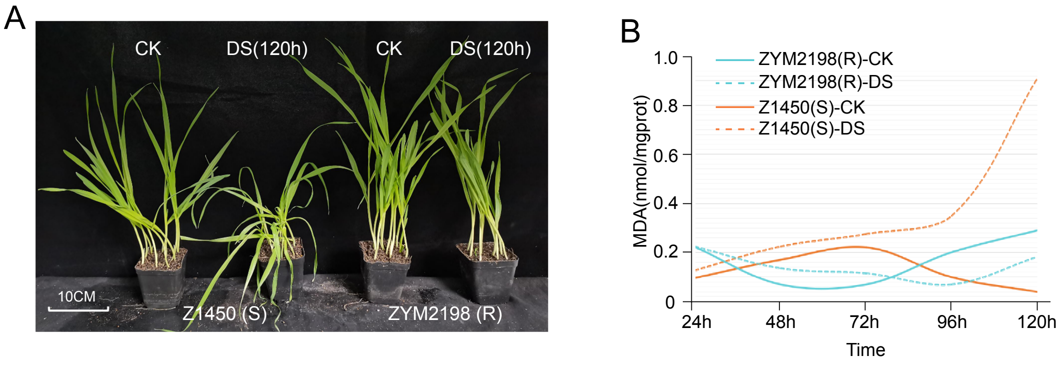

Supplemental Figure 1. Wilting phenotype and verification of drought sensitive and drought resistant qingke. **(A)** Wilting of drought-sensitive qingke Z1450 and drought-resistant qingke ZYM2198 before and after 120 hours of drought stress at seedling stage; scale bar, 10 cm. **(B)** Malondialdehyde (MDA) content in leaves of drought-resistant qingke ZYM2198 (blue) and drought-sensitive qingke Z1450 (orange) under control (solid) or different drought stress (dashed) time.

Supplemental Figure 2

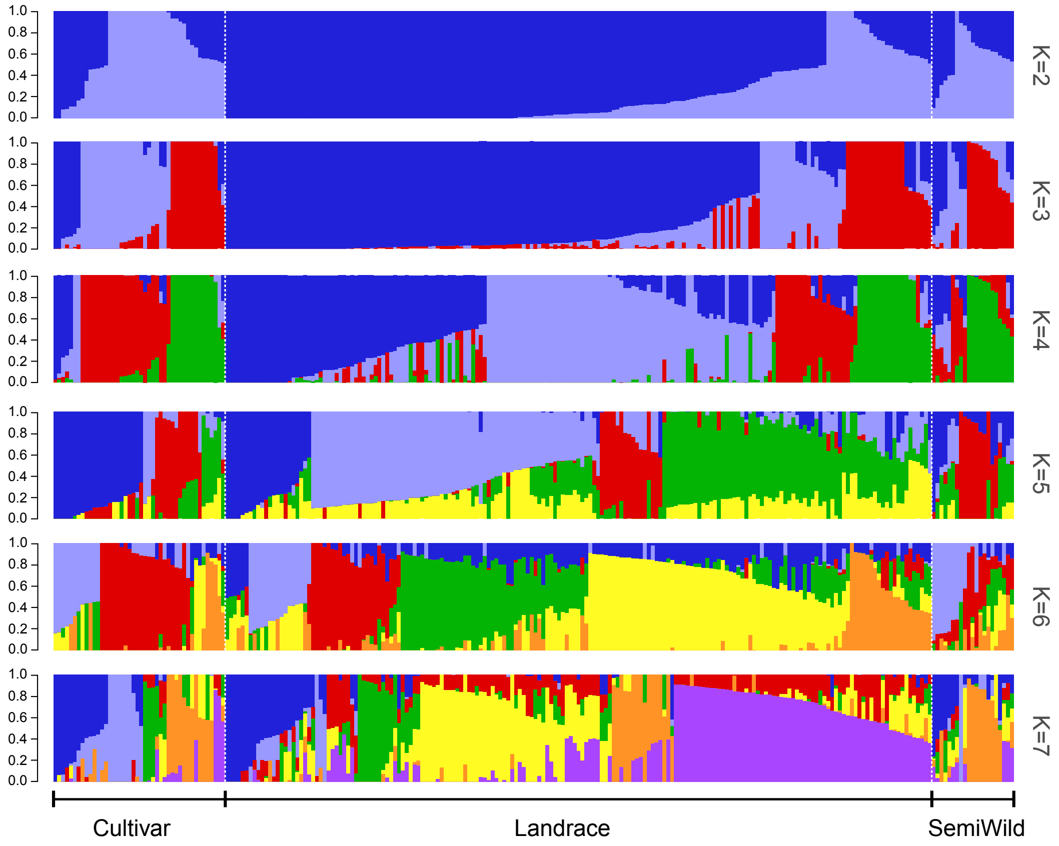

Supplemental Figure 2. Population structure in the ADMIXTURE analysis at K=2 to K=7.

Supplemental Figure 3

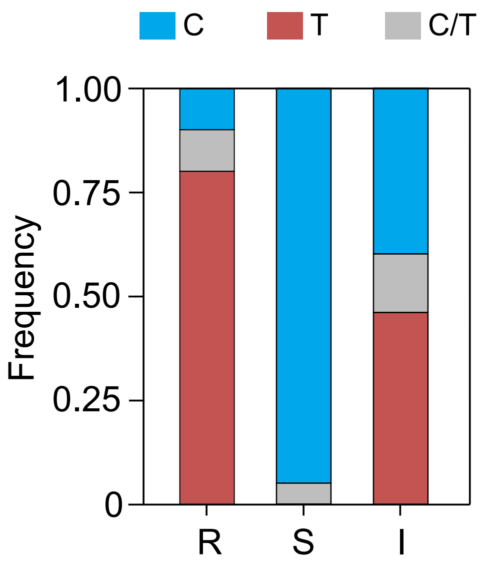

Supplemental Figure 3. The base frequency of SNP chr2H:652503665 in drought-resistant (R), drought-sensitive (S) and intermediate (I) group of qingkes, and the bases represented by different colors are consistent with the (F).

Supplemental Figure 4

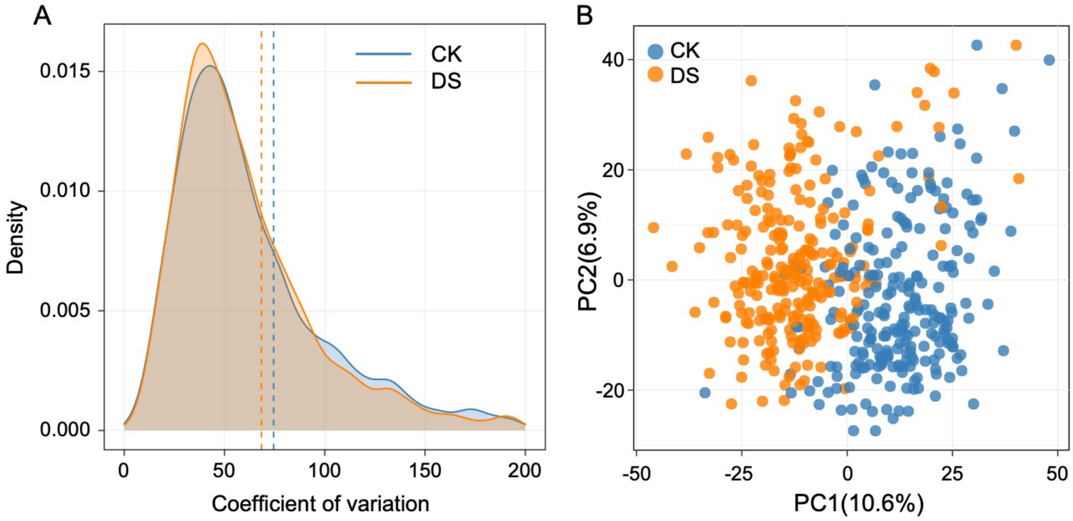

Supplemental Figure 4. CV and PCA analysis of metabolites in qingke leaves under CK and DS conditions. **(A)** Density distribution of CV values of metabolites under CK (blue) or DS (orange) conditions. **(B)** Principal component analysis (PCA) score plot showing distinctive metabolic profiles of all accession of qingke under CK (orange) or DS (blue) condition.

Supplemental Figure 5

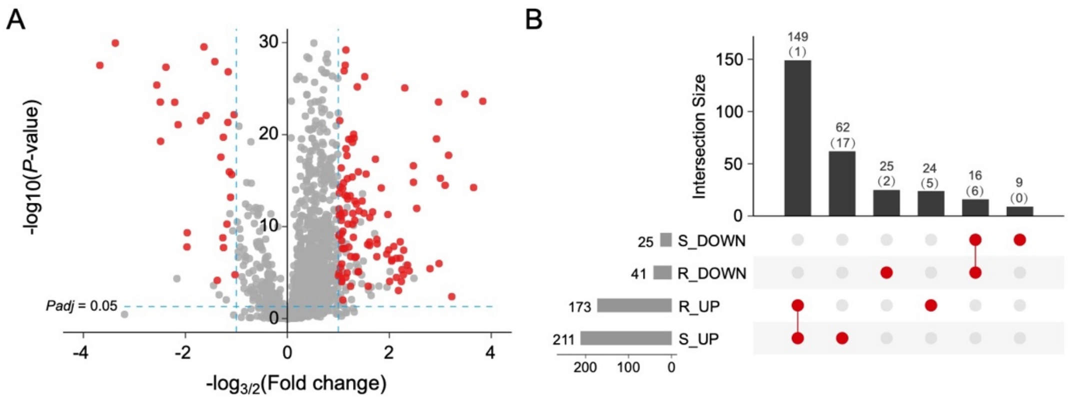

Supplemental Figure 5. Analysis of differential accumulation of metabolites in response to drought stress in qingke. **(A)** A volcano plot of the metabolic differences in the qingke leaf after 48h of drought stress. The red dot represents a metabolite with a fold change>=1. or <0.67, *P*-value < 0.05 and VIP>1. **(B)** The UpSet plot represents the intersection between the sets of up-regulated (UP) or down-regulated (DOWN) metabolites of drought-resistant (R) and drought-sensitive (S) qingke in response to drought stress. The vertical bar plot reports the intersection size, the dot plot reports the set participation in the intersection, and the horizontal bar plot reports the set sizes.

Supplemental Figure 6

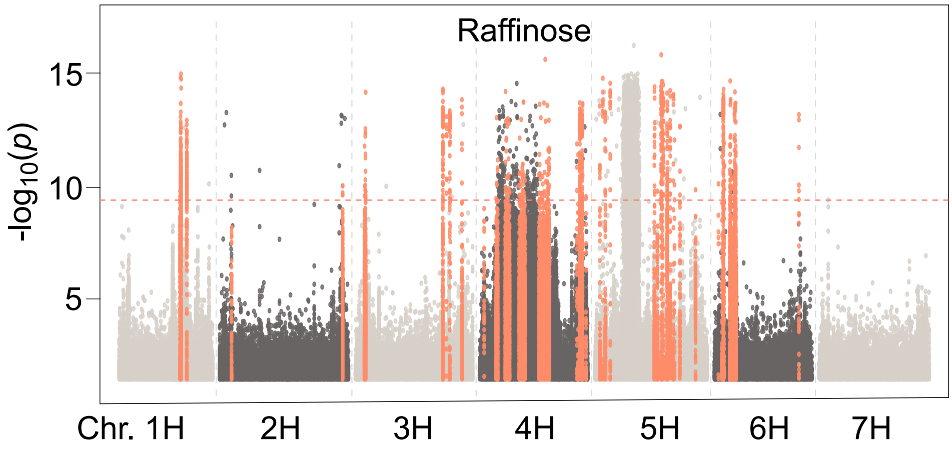

Supplemental Figure 6. Manhattan plot displaying the mGWAS results for the content of raffinose in DS condition. Orange dots indicate SNP in significance association loci.

Supplemental Figure 7

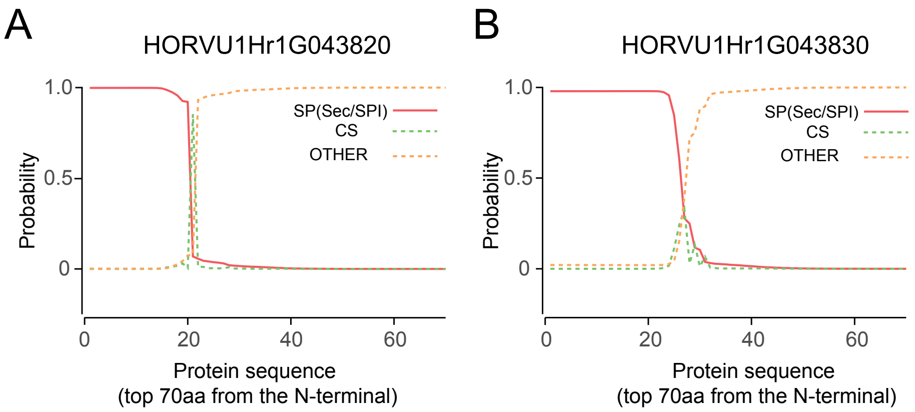

Supplemental Figure 7. (A-B) Sequence analysis of the HORVU1Hr1G043820 (A) and HORVU1Hr1G043830 (B) signal peptide by SignalP 5.0 server (http://www.cbs.dtu.dk/services/SignalP), the first 70 amino acids in the N-terminal were used.

Supplemental Figure 8

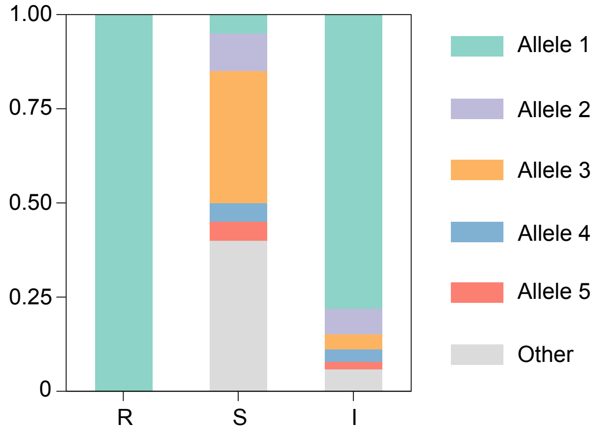

Supplemental Figure 8. The base frequency of alleles described in Figure 5E at HORVU1Hr1G043820 and HORVU1Hr1G043830 genes in drought-resistant (R), drought-sensitive (S) and intermediate (I) group of qingkes.

Supplemental Figure 9

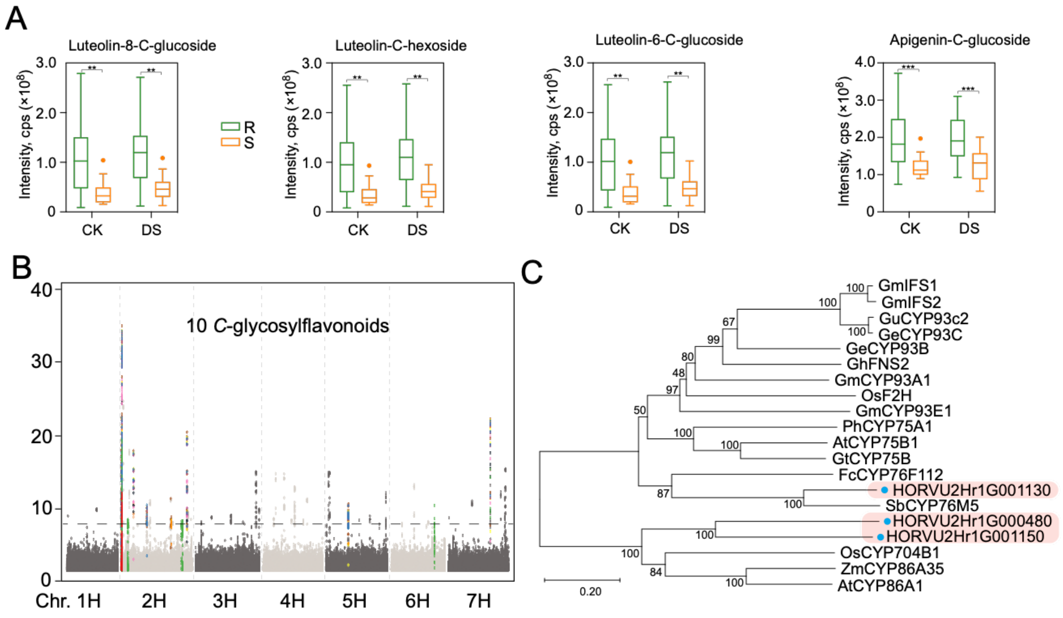

Supplemental Figure 9. Functional analysis of constitutive metabolites related to drought resistance and its candidate genes in qingke. **(A)** The boxplot represents the other four C-glycosylflavonoids with higher accumulation in drought-resistant qingke under CK and CS condition. The asterisks represent a significant difference of Students't test at *P*-value < 0.05 (*), *P*-value < 0.01 (**) or *P*-value < 0.01 (***). **(B)** Manhattan plot displaying the mGWAS co-location of 10 C-glycosylflavonoids in DS condition. Colored dots indicate SNP in significance association loci. **(C)** An unrooted phylogenetic tree of CYP450 genes was constructed as described in Methods. Bootstrap values (based on 1000 replications) are indicated at each node (bar: 0.2 amino acid substitutions per site).

Supplemental Figure 10

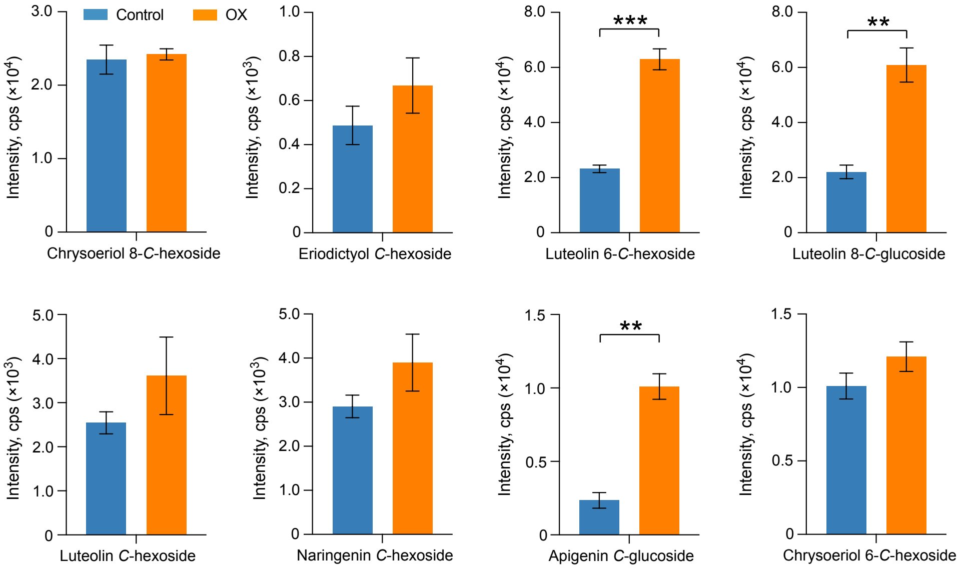

Supplemental Figure 10. Accumulation of chrysoeriol 8-C-hexoside, eriodictyol C-hexoside, luteolin 6-C-hexoside, luteolin 8-C-glucoside, luteolin C-hexoside, naringenin C-hexoside, apigenin C-glucoside and chrysoeriol 6-C-hexoside in tobacco leaves transiently overexpressing HORVU2Hr1G001460. The asterisk represent a significant difference of Students't test at *P*-value < 0.01 (**) or *P*-value < 0.001 (***) between CK and overexpression line (OX). Data are shown as the mean ± SEM, n = 3. Data are shown as the mean ± SD, n = 3.

Supplemental Figure 11

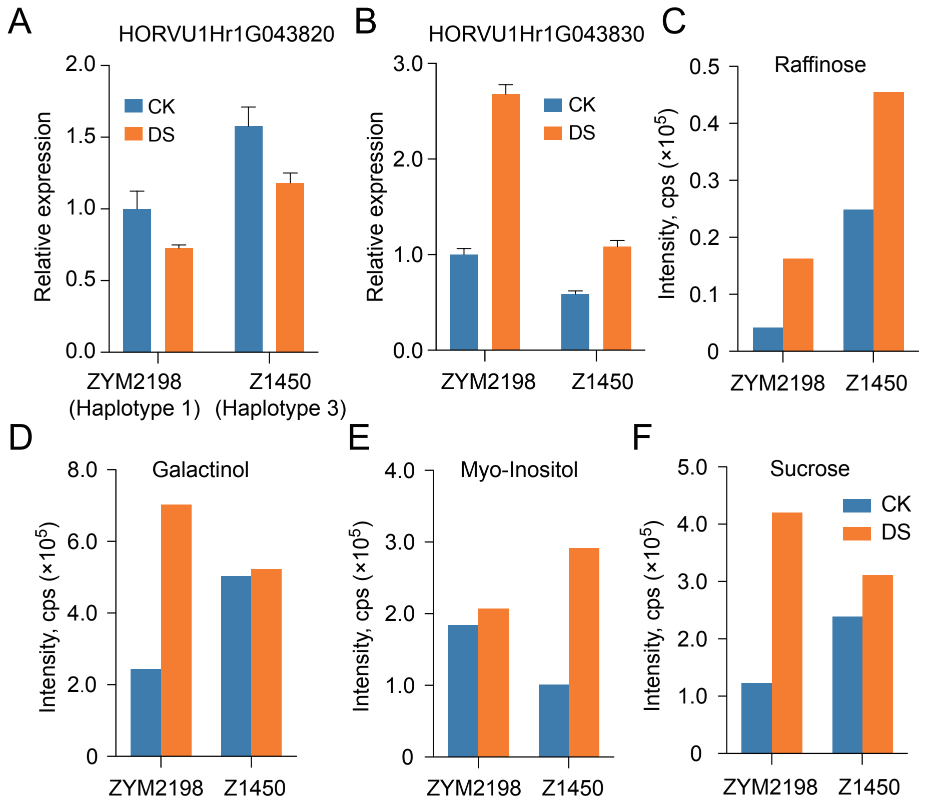

Supplemental Figure 11. Content of raffinose related metabolites and candidate gene expression of raffinose in ZYM2198 and Z1450. **(A)** Relative expression of HORVU1Hr1G043820 in drought-resistant qingke (ZYM2198) and drought-sensitive qingke (Z1450) under CK and DS conditions, *HvADP-RF 1* as internal control. **(B)** Relative expression of HORVU1Hr1G043830 in drought-resistant qingke (ZYM2198) and drought-sensitive qingke (Z1450) under CK and DS conditions, *HvADP-RF* as internal control. Data are shown as the mean ± SEM, n = 3. **(C-F)** The contents of raffinose, galactinol, myo-inositol, sucrose in ZYM2198 and Z1450 under CK and DS conditions.

Supplemental Figure 12

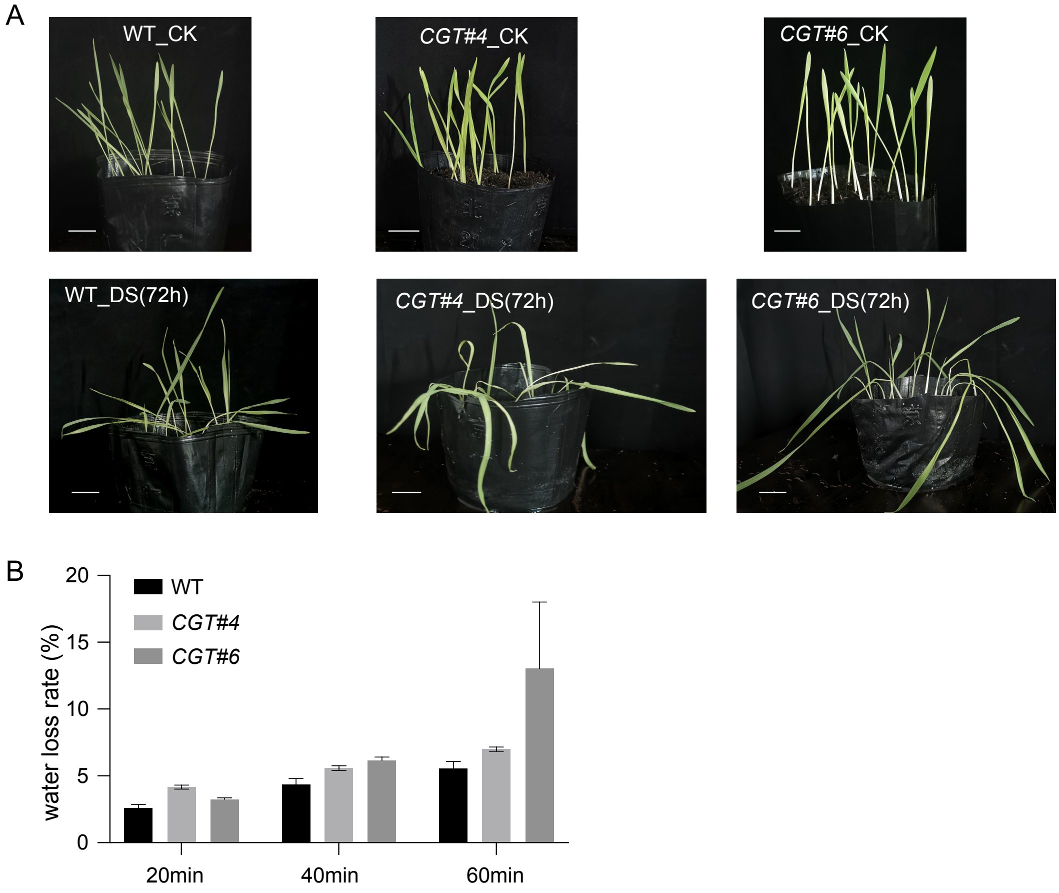

Supplemental Figure 12. Phenotypes of *CGT* gene mutants by EMS mutagenesis. **(A)** The screened *CGT* gene mutants (*CGT#4, CGT#6*) and their corresponding parent varieties (landrace "Hatiexi", WT) were displayed in the greenhouse pot under the optimal conditions (CK) or after 72 hours of drought stress (DS), respectively. **(B)** Water loss rate of leaves of *CGT* gene mutants (*CGT#4, CGT#6*) and their corresponding parent varieties (landrace "Hatiexi", WT) in air environment after 20min, 30min and 60min. Data are shown as the mean ± SEM, n = 6.
